# A highly expressed miR-101 isomiR is a functional silencing small RNA

**DOI:** 10.1186/1471-2164-14-104

**Published:** 2013-02-15

**Authors:** Franc Llorens, Mónica Bañez-Coronel, Lorena Pantano, Jose Antonio del Río, Isidre Ferrer, Xavier Estivill, Eulàlia Martí

**Affiliations:** 1 Molecular and Cellular Neurobiotechnology Group, Institut de Bioenginyeria de Catalunya (IBEC), Parc Científic de Barcelona, Barcelona, Spain; 2 Department of Cell Biology, University of Barcelona (UB), Barcelona, Spain; 3 Network Biomedical Research Center for Neurodegenerative Diseases (CIBERNED), Barcelona, Spain; 4 Genetic Causes of Disease Group, Genes and Disease Program, Centre for Genomic Regulation (CRG) and UPF, Barcelona, Spain; 5 Universitat Pompeu Fabra (UPF), Barcelona, Spain; 6 Centro de Investigación Biomédica en Red de Epidemiología y Salud Pública, (CIBERESP), Barcelona, Spain; 7 Institut de Medicina Predictiva i Personalitzada del Càncer, Badalona, Spain; 8 Institut Neuropatologia, Servei Anatomia Patològica, IDIBELL-Hospital Universitari de Bellvitge, Universitat de Barcelona, Barcelona, Spain

**Keywords:** MicroRNA, miR-101, IsomiR, Ultra-sequencing, Deep-sequencing

## Abstract

**Background:**

MicroRNAs (miRNAs) are short non-coding regulatory RNAs that control gene expression usually producing translational repression and gene silencing. High-throughput sequencing technologies have revealed heterogeneity at length and sequence level for the majority of mature miRNAs (IsomiRs). Most isomiRs can be explained by variability in either Dicer1 or Drosha cleavage during miRNA biogenesis at 5’ or 3’ of the miRNA (trimming variants). Although isomiRs have been described in different tissues and organisms, their functional validation as modulators of gene expression remains elusive. Here we have characterized the expression and function of a highly abundant miR-101 5’-trimming variant (5’-isomiR-101).

**Results:**

The analysis of small RNA sequencing data in several human tissues and cell lines indicates that 5’-isomiR-101 is ubiquitously detected and a highly abundant, especially in the brain. 5’-isomiR-101 was found in Ago-2 immunocomplexes and complementary approaches showed that 5’-isomiR-101 interacted with different members of the silencing (RISC) complex. In addition, 5’-isomiR-101 decreased the expression of five validated miR-101 targets, suggesting that it is a functional variant. Both the binding to RISC members and the degree of silencing were less efficient for 5’-isomiR-101 compared with miR-101. For some targets, both miR-101 and 5’-isomiR-101 significantly decreased protein expression with no changes in the respective mRNA levels. Although a high number of overlapping predicted targets suggest similar targeted biological pathways, a correlation analysis of the expression profiles of miR-101 variants and predicted mRNA targets in human brains at different ages, suggest specific functions for miR-101- and 5’-isomiR-101.

**Conclusions:**

These results suggest that isomiRs are functional variants and further indicate that for a given miRNA, the different isomiRs may contribute to the overall effect as quantitative and qualitative fine-tuners of gene expression.

## Background

miRNAs are small non-coding RNAs that generally act as negative post-transcriptional regulators of gene expression. It is estimated that miRNAs regulate at least 20% of human genes [1], constituting an important layer of regulation in gene expression networks. The influence of a particular miRNA on the transcriptome is difficult to dissect, since genes can harbor binding sites for several miRNAs, and a specific miRNA can modulate the expression of hundreds of mRNA targets [2]. miRNAs are involved in almost every biological process examined and their deregulation has been associated with a number of pathological conditions, including cancer and neurological disorders [3-6].

Mature miRNAs function as components of the RNA-Induced Silencing Complex (RISC) [7], guiding the RISC to specific gene targets through base-pairing interactions between the miRNA and the mRNA. Members of the Argonaute (Ago) protein family are central to RISC function. The seed region extends from the second to the seventh or eighth position of mature miRNA and has traditionally been considered as a major determinant of the target mRNA repertoire, through perfect base pairing with the 3’-untranslated regions (3’-UTR) of mRNAs [8]. Other structures of the 3’-end of the miRNA are also involved in duplex stability, providing a supplementary site if the seed is fully paired or a compensatory site when the 5’-end has mismatches or bugles [9,10]. However, our knowledge of the determinants governing gene targeting is far from complete. In fact, recent findings show that targeting can occur through sites other than the 3’-UTR, and that seed region perfect base pairing is not always required [11-14]. This miRNA/mRNA binding leads either to target mRNA degradation or to translational inhibition, depending, among other factors, on the extent of base-pairing and the type of RISC/Ago proteins [15].

A novel degree of complexity for miRNA transcriptome has been highlighted using next generation sequencing (NGS) strategies. NGS has revealed post-transcriptional editing processes in miRNAs, mainly consisting in variations in the 3’- and 5’-terminus and to a minor extent, nucleotide substitutions along the miRNA sequence [16-19]. Variations at the miRNA ends may be generated by a shift in Drosha and Dicer cleavage sites during miRNA biogenesis (trimming variants) [19,20]. Another type of variability is related to pri-miRNA post-transcriptional editing as a consequence of adenosine or cytidine deaminase activity (nucleotide substitution variants). Finally, nucleotide additions at the 3’-end of the miRNA are another source of miRNA variability [21-23]. The resulting sequences differ slightly from the annotated miRNA and have been termed isomiRs [19].

Several lines of evidence suggest that isomiRs are not experimental artifacts derived from RNA degradation during sample preparation for NGS [24,25]. In fact the frequencies of nucleotide substitutions are very diferent from sequencing error rates; and the detection of isomiRs in many species, tissues and physiological conditions [26,27], suggests that they may have a biological role. This notion is further supported by their differential expression across developmental stages [27,28]. This scenario suggests that, for a given miRNA, posttranscriptional gene expression may depend on different isomiRs. However, their functionality as posttranscriptional gene expression regulators needs to be proven. Our recent studies of miRNA profiling in human brain samples have shown a multitude of miRNA variants [16]. miR-101 is an interesting example, since it presents 5’-trimming isomiRs (5’-isomiR-101) expressed at levels similar to those of the reference sequence. Here, we characterize the ability of this highly expressed human isomiR-101 to work as a functional miRNA.

## Results

### miR-101 and miR-101 5’-isomiRs are expressed at different proportions in different human samples

**Figure 1:**
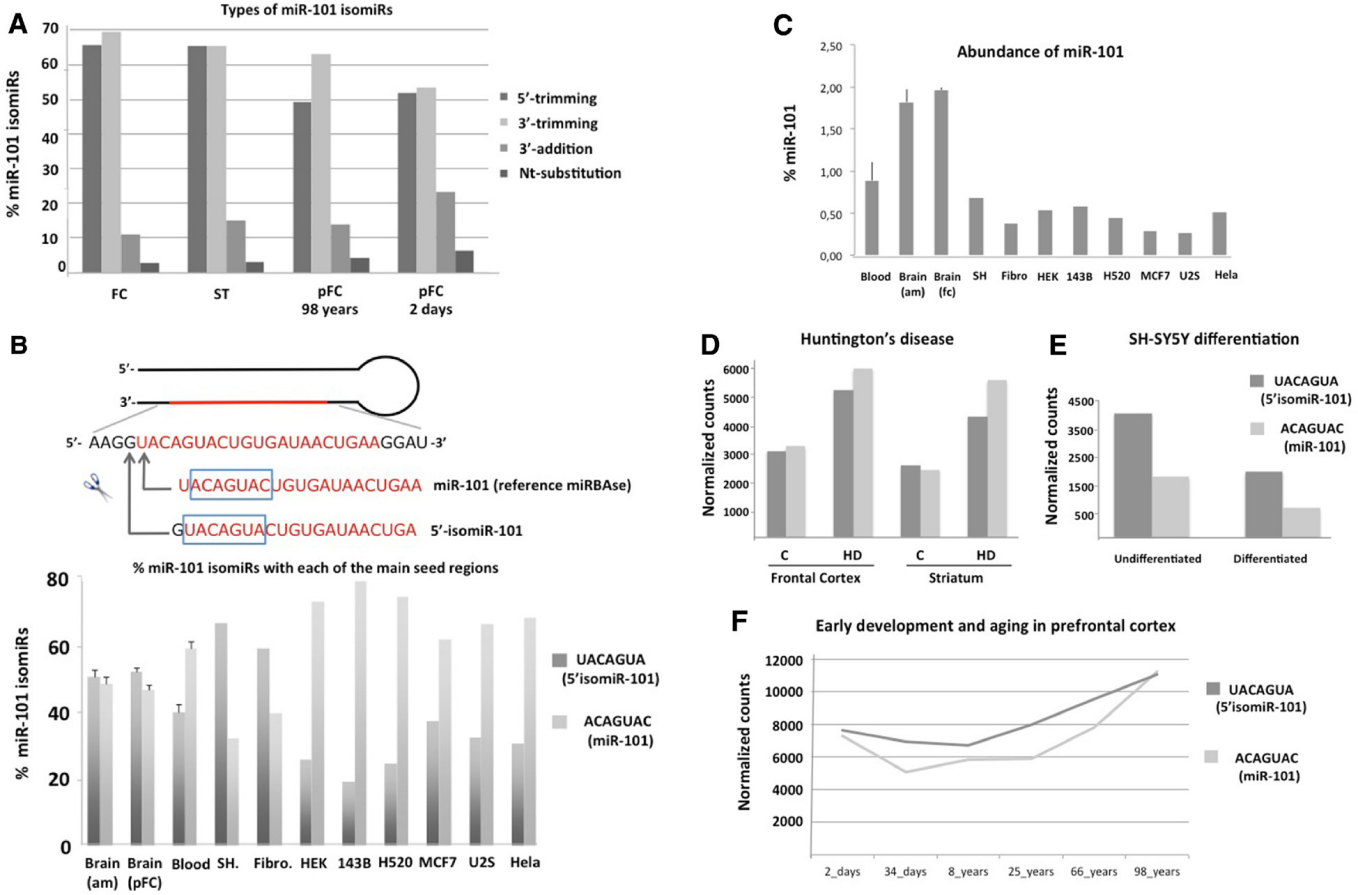
**miR-101 and 5’-isomiR-101 are abundant in different cells and tissues. A.** Relative frequencies of different miR-101 isomiRs in the frontal cortex (FC) and striatum (ST) of a control individual [16] and prefrontal cortex (pFC, 98 years and 2 days old) [26]. The summed frequency of all sequences mapping onto miR-101 was considered as the 100%. **B.** Percentage of each of the two main seed regions in human brain (4 individuals) and blood (4 individuals), and human cell lines [29]. A scheme is included showing miR-101 precursor and mature forms (in red). The arrows point to the major cleavage sites producing the most abundant mature miR-101 variants. The seed regions in each variant are contained in a blue box. **C.** Abundance of miR-101 in human blood and brain (amygdala –am- of 4 individuals and frontal cortex –fc- of two individuals aging 25 and 66 years [26]) and in cell lines [29]. In each sample, the percentage of sequences mapping onto miR-101 is calculated with respect to the total of sequences mapping onto miRNA database. **D-F.** miR-101 and 5’-isomiR-101 expression in the frontal cortex and the striatum of control individuals (**C**) and patients with HD [16]; in undifferentiated and differentiated SH-SY5Y cells and in the frontal cortex of individuals at different ages [26]. In **D**, **E**, **F** normalized counts are expressed as the ratio: (frequency of sequences presenting miR-101 or 5’isomiR-101 seed regions) / (frequency of sequences mapping onto miRNAs) * 10E6. When more than a biological replica is available (**B** and **C**) data are presented as the mean ± standard deviation. SH: SH-SY5Y human neuroblastoma cell line, Fibro: Fibrocytes, HEK: human embryonic kidney 293 cells, 143B: human bone osteosarcoma 143B cell line, H520: human squamous cell carcinoma H520 cell line, MCF7: human breast cancer MCF7 cell line, U2S: human osteosarcoma U2S cell line, HeLa: human cervical cancer HeLa cell line.

We have shown that the vast majority of miRNAs in human frontal cortex and striatum display sequence variants or isomiRs [16]. Several factors may modulate the physiological relevance of an isomiR, including i) the amount of the isomiR in relation to the rest of sequences mapping onto the same miRNA locus and ii) the type of variant, with special emphasis on nucleotide changes affecting the seed region. Sequencing data analysis of these brain areas showed that miR-101 was an abundant miRNA, represented by approximately 100 unique isomiRs (> 10 counts per sequence), although deeper sequencing performed in other brain samples identified more variants (Additional file 1: Figure S1, Additional file 2: Table S1). The different miR-101 isomiRs consisted in trimming variants, nucleotide additions at the 3’ end of the miRNA and nucleotide substitutions at different positions along the mature miRNA (Additional file 1: Figure S1, Additional file 2: Table S1, Figure 1A). The distribution of the different types of miR-101 isomiRs was confirmed in the prefrontal cortex of human brains at different ages in an independent sequencing experiment [26] (Figure 1A). Trimming variants affecting the 5’ end of the miRNA (5’-isomiRs) were among the more abundant species. In fact, the vast majority of miR-101 5’-isomiRs affected a single nucleotide upstream of the reference miR-101, therefore defining a new seed region. Thus, taking into consideration all the sequences mapping onto miR-101, two main types of seeds were found: ACAGUAC, which is reported in the miRBase, and UACAGUA, corresponding to the vast majority of miR-101 5’-isomiRs. We examined the presence of miR-101 5’-isomiRs in another brain area (amygdala) from four individuals without major histopathological lesions and no clinical neuropathology and in peripheral blood from four healthy individuals, and further analyzed several public high-throughput sequencing datasets from the GEO repository corresponding to human brains at different developmental stages [26] and different human cell lines [29] (Figure 1B). This analysis indicated that miR-101 5’-isomiRs are expressed at high proportions in different human cells, thus suggesting a physiological role for these variants. Furthermore, the proportion of each of the major seed regions differs according to the cell type, which suggests a differential modulation of the biogenesis and/or stability of the different miR-101 variants.

Considering all the sequencing datasets examined, the percentage of sequences mapping onto miR-101 varied between tissues and cell lines, with the brain presenting the highest levels (Figure 1C). These data suggest that high miR-101 levels may be important for adult brain physiology. In fact, miR-101 expression is regulated under a number of physiological and pathological conditions, including angiogenesis [30], tumors [31,32] and neurodegeneration [16,33,34]. In the last condition, our earlier study identified miR-101 as an up-regulated gene in HD [16]. However, that study was an overall analysis of all the sequences mapping onto miR-101. To discern the specific expression pattern of miR-101 5’-isomiRs, we evaluated the expression of miR-101 variants harboring each of the two main seeds in control brains and brains of patients with HD (Figure 1D). Both types of variants were up-regulated in the frontal cortex and striatum of HD patients. In addition, we performed a similar analysis in other physiological conditions of the nervous system, including *in vitro* differentiation of the SH-SY5Y neuronal cell line to a postmitotic dopaminergic phenotype (Figure 1E) and early postnatal development and ageing of the human brain [26] (Figure 1F). Interestingly, the relative amounts of both types of miR-101 variants in the brain differed according to age. These data suggest that miR-101 is modulated in the development and differentiation processes of the nervous system. Furthermore, the context-dependent variations in the relative amounts of miR-101 and 5’-isomiR-101 suggest that the mechanisms regulating their biogenesis and/or stability may differ depending on the biological process.

### miR-101 and 5’-isomiR-101 interact with Ago2 and Rck/p54

Functional studies aiming to elucidate the possible relevance of the more abundant miR-101 isomiRs in the nervous system were performed in the neuronal cell line SH-SY5Y. We used miRNA mimics for two abundant representative sequences containing ACAGUAC or UACAGUA seeds: The miRBase reference miR-101 UACAGUACUGUGAUAACUGAA, and one of the most abundant miR-101 5’-isomiRs presenting the same length as the reference miR-101 GUACAGUACUGUGAUAACUGA (Additional file 1: Figure S1).

**Figure 2:**
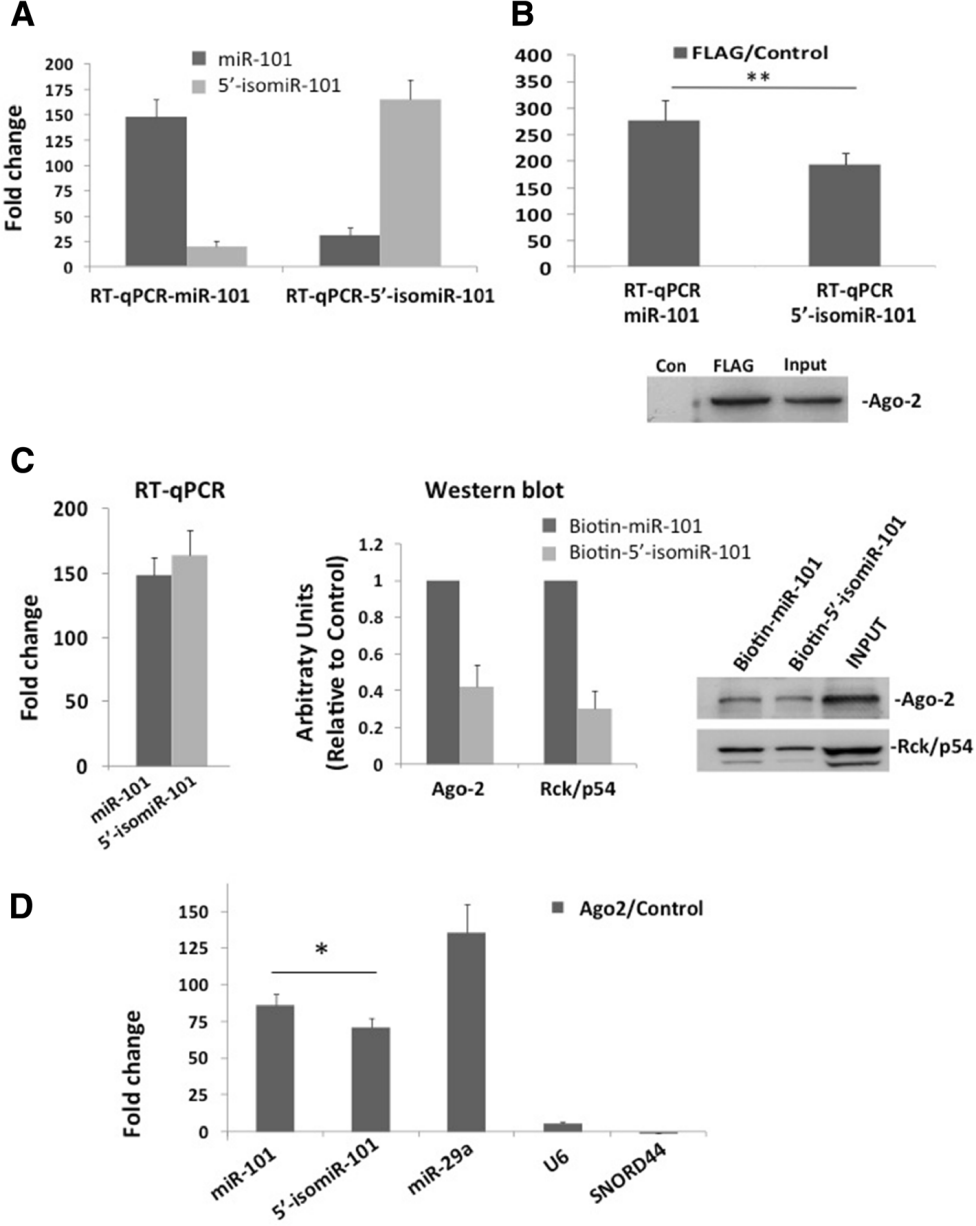
**miR-101 and 5’-isomiR-101 bind Ago2 and Rck/p54. A.** Specificity of miR-101 and 5’-isomiR-101 detection. SH-SY5Y cells were transfected with a negative control sequence (siGLO Green) or the mIRIDIAN mimics for miR-101 (dark bars) or 5’-isomiR-101 (light bars). Specific RT-qPCRs determinations for miR-101 and 5’-isomiR-101 were performed. Results are expressed as the Fold Change ratios (miridian mimic / control siGLO). **B.** Expression of exogenously transfected miR-101 or 5’-isomiR-101 in Ago2 immunocomplexes. SH-SY5Y cells were transfected with Siglo Green or the mIRIDIAN mimics for miR-101 or 5’-isomiR-101. Specific RT-qPCRs were performed in Ago2-FLAG immunoprecipitates.Results are expressed as the Fold Change ratios obtained from the FLAG antibody/Control antibody immunoprecipitates. Western-blot with anti-FLAG antibody shows the presence of Ago2-FLAG in the FLAG-IP and input, and the absence of Ago2-FLAG in the control IP. **C.** Detection of RISC components in miR-101 or 5’-isomiR-101 pull-down. SH-SY5Y cells stably expressing Ago2-FLAG were transfected with mIRIDIAN 3’-biotin-miR-101 or 3’-biotin-5’-isomiR-101. Biotin-miRNA complexes were pulled down with streptavidin beads and subsequently assayed for western blot analysis using Ago2 and Rck/p54 antibodies. The relative amount of Ago2 and Rck/p54 in 3’-biotin-5’-isomiR-101 versus 3’-biotin-miR-101 is shown in the middle panel. miRNA-101 transfected samples were used to normalize data and were assigned a value of 1. Left panel shows similar amounts of transfected 3’-biotin-miR-101 or 3’-biotin-5’-isomiR-101 determined in total cell extracts with the corresponding specific RT-qPCR assays. **D.** Expression of endogenous miR-101 and 5’isomiR-101 in Ago2-immunocomplexes in the human brain. Ago2 complexes were immunoprecipitated from human frontal cortex homogenates and miR-101, 5’-isomiR-101, miR-29a, U6 SNORD44 were determined by RT-qPCR. Results are expressed as the Fold Change ratios obtained from the Ago2 *vs* denatured Ago2 immunoprecipitates. Three independent experiments were performed in **A-D**. All data are expressed as the mean± SEM. Asterisks indicate statistical significance between miR-101 and 5’-isomiR-101 data : * (p ≤ 0.05), ** (p≤0.01) using Mann–Whitney test.

We used highly specific LNA™ RT-qPCR assay (Exiqon™) for selective detection of miR-101 or 5’-isomiR-101. To test the specificity of the detection, we transfected equivalent amounts of either miR-101 or 5’-isomiR-101 mature mIRIDIAN-mimics or a control sequence into SH-SY5Y cells and determined the amount of miR-101 and 5’-isomiR-101 using the corresponding assays (Figure 2A). Fold changes are expressed relative to the cells transfected with the control sequence. The miR-101 RT-qPCR specific assay clearly detected miR-101 while 5’-isomiR-101 was very weakly amplified (Figure 2A, left bars). A complementary result was obtained using the 5’-isomiR-101 RT-qPCR specific assay (Figure 2A, right bars), indicating that both assays are highly selective for each variant.

We then examined whether transfected miR-101 and 5’-isomiR-101 mIRIDIAN were incorporated into the RISC, using SH-SY5Y cells stably expressing Ago2-FLAG. We performed immunoprecipitation (IP) assays using anti-FLAG antibodies for Ago2-IP or control antibodies, and subsequently RNA in the IP was isolated. We detected a significant enrichment of either miR-101 or 5’-isomiR-101 in Ago2 IP. The amount of miR-101 or 5’-isomiR-101 in cells transfected with the corresponding miRNA mimics was similar (Figure 1A). However, increased amounts of miR-101 were detected in the Ago2 IP, compared with 5’-isomiR-101 (Figure 2B), suggesting that miR-101 was more efficiently loaded into RISC.

The ability of miR-101 and 5’-isomiR-101 to bind to the RISC was further confirmed in complementary experiments designed to evaluate the presence of RISC members in transfected biotinylated miR-101 and 5’-isomiR-101 (Figure 2C). mIRIDIAN 3’-biotin-miR-101 and 3’-biotin-5’-isomiR-101 were transfected in SH-SY5Y cells and then pulled down using streptavidin beads. The samples were blotted against Ago2 and Rck/p54 which represses translation and associates with the RISC complex in order to regulate miRNA mediated gene silencing [35]. Both biotin-miR-101 and biotin-5’-isomiR-101 were able to interact with Ago2 and Rck/p54. Although similar levels of each biotinylated mIRIDIAN were detected in transfected SH-SY5Y cells, increased levels of both proteins were found in biotin-miR-101 pull downs compared with those of biotin-5’-isomiR-101. This result suggests that miR-101 binds with RISC members more efficiently, which is in agreement with results shown in Figure 1B.

To validate the presence of both variants in endogenous RISC, we immunoprecipitated Ago2 from the human frontal cortex. The control experiment included a sample in which the Ago2 antibody had previously been denaturized (d-Ago2) and was therefore unable to interact with Ago2. Total RNA was isolated from Ago2 and d-Ago2 IPs and subsequently we performed RT-qPCR for miR-101, 5’-isomiR-101, miR-29a, SNORD44 and U6. Data were expressed as the fold change obtained from the Ago2 vs d-Ago2 crossing thresholds (CTs) (Figure 2D). miR-101 and 5’-isomiR-101 were enriched in Ago2 IPs compared to control d-Ago2 antibody. miR-29a, a highly abundant miRNA in brain [16] was also enriched in Ago2 complexes. In contrast, the small nucleolar RNAs U6 and SNORD44, which were not expected to bind Ago2, were detected at insignificant levels. The presence of both types of miR-101 variants in Ago2 immunocomplexes was confirmed in publicly available high-throughput sequencing data of endogenous Ago2-associated sRNAs in HEK293T and mouse NIH-3T3 cells (GEO datasets GSM337571 and GSM849857 respectively).

To evaluate the possibility that 5’-isomiR-101 and miR-101 are sorted into different Agos, we analyzed publicly available small-RNA sequencing datasets from Ago1, Ago2 and Ago3 IP and total cell extracts, in THP-1 human monocytic cells [36] (Additional file 3: Figure S2). We found all types of miR-101 in each Ago (the trimming, substitution and 3’-additions variants). In addition, the percentage of miR-101 sequences loaded onto each Ago was comparable. Grouping the variants according to the two main seeds we found that in total cell homogenates the ratio miR-101/5’-isomiR-101 in THP-1 cells was close to 2. However, this ratio varied slightly depending on the Ago protein, with the highest levels being found in Ago2 and the lowest in Ago3. This suggests that although in terms of quantity miR-101 exceeded 5’-IsomiR-101 in all Agos, miR-101 species loaded more onto Ago2 than 5’-isomiR-101 variants. This is in agreement with our findings for SH-SY5Y.

### miR-101 and 5’-isomiR-101 differentially repress COX-2, Mcl-1, APP, EZH2 and MKP-1 expression

Since miR-101 and 5’-isomiR-101 have a different seed region, we evaluated the predicted conserved targets for each variant, using the TargetScan 5.2 release [37], by searching for the presence of 8mer and 7mer sites that match miR-101 and 5’-isomiR-101 seed regions. TargetScan 5.2 detected that the majority of putative mRNA targets (>70%) were coincidental, suggesting that miR-101 and 5’-isomiR-101 may target highly overlapping pathways. In an attempt to identify possible differentially deregulated genes, SH-SY5Y cells were transfected with miR-101 or 5’-isomiR-101 mimics or a scrambled mimic in three independent experiments, and 48 h later overall gene expression was evaluated using Illumina microarrays (HumanHT Expression BeadChips). Transfection of each miR-101 variant resulted in few significantly deregulated genes that showed few changes in expression (q<5%, fold change < −1,2 or > 1,2; Additional file 4: Table S2–Additional file 5: Table S3). miR-101 induced a significant downregulation of 16 genes, eight being targeted by at least one prediction algorithm. Nine out of the 16 genes were also downregulated by 5’-isomiR-101 (q<10%), while the rest did not show significant variations. Thus, 5’-isomiR-101 seems to induce an overall weaker inhibition. In agreement, transfection of 5’-isomiR-101 resulted in a slight downregulation of two genes, for which miR-101 did not induce significant expression changes. These data indicate that under our experimental conditions modulation of gene expression by miR-101 or 5’-isomiR-101 may not involve mRNA degradation as the main mechanism, and suggest further that miR-101 inhibits gene expression more efficiently than 5’-isomiR-101.

To gain insights into the possible role of 5’isomiR-101 in physiological conditions, we analyzed publicly available mRNA and small-RNA datasets of human brain samples at multiple time-points between early postnatal development and aging [38]. We chose this paradigm for two reasons: i/ miR-101 expression levels increased through aging, suggesting a role in this process (Figure 1F), and ii/ variants carrying each seed are not equally expressed at the different time-points (compare the oldest and the middle-aged individuals, Figure 1F), suggesting a seed-specific role in the modulation of the age-related genes.

To assess the possible seed-specificity in the modulation of the age-related transcriptome, we analyzed the correlation between the expression of miR-101 variants and that of the age-related genes at different ages through life. First, we generated a list of candidate age-related genes through a multiple regression analysis of the gene expression profiles using age as the predicted variable (a total of 3801). We then identified the miR-101 or 5'-isomiR-101 predicted targets among the pool of age-related genes, using the TargetScan 5.2 algorithm (837 for miR-101 and 899 for 5’-isomiR-101, out of the 3801; the majority, 73%-78%, were common to the two miR-101 seeds). Finally, we measured the correlation between the expression levels of age-related genes and those of the most abundant reference seed sequence (miR-101, UACAGUACUGUGAUAACUGAA) or the most abundant 5’-isomiR sequence (5’-isomiR-101, GUACAGUACUGUGAUAACUGA).

The negative correlation between the expression profiles of the mRNAs and those of miR-101 or 5’-isomiR-101 was considered the readout for miR-101 or 5’-isomiR-101 effective/detectable gene targeting. The expression of a total of 64 and 153 age-related genes anti-correlated with that of the ref-miR-101 or 5’-isomiR-101 respectively (r<−0.7), with a significantly lower number of common genes between the two miR-101 seeds (16%-39%, p<0.01). These results indicate that the majority of anti-correlated targets were seed-specific, which supports the notion of a selective function of each miR-101 variant in modulating age-related genes (Additional file 6: Figure S3).

**Figure 3:**
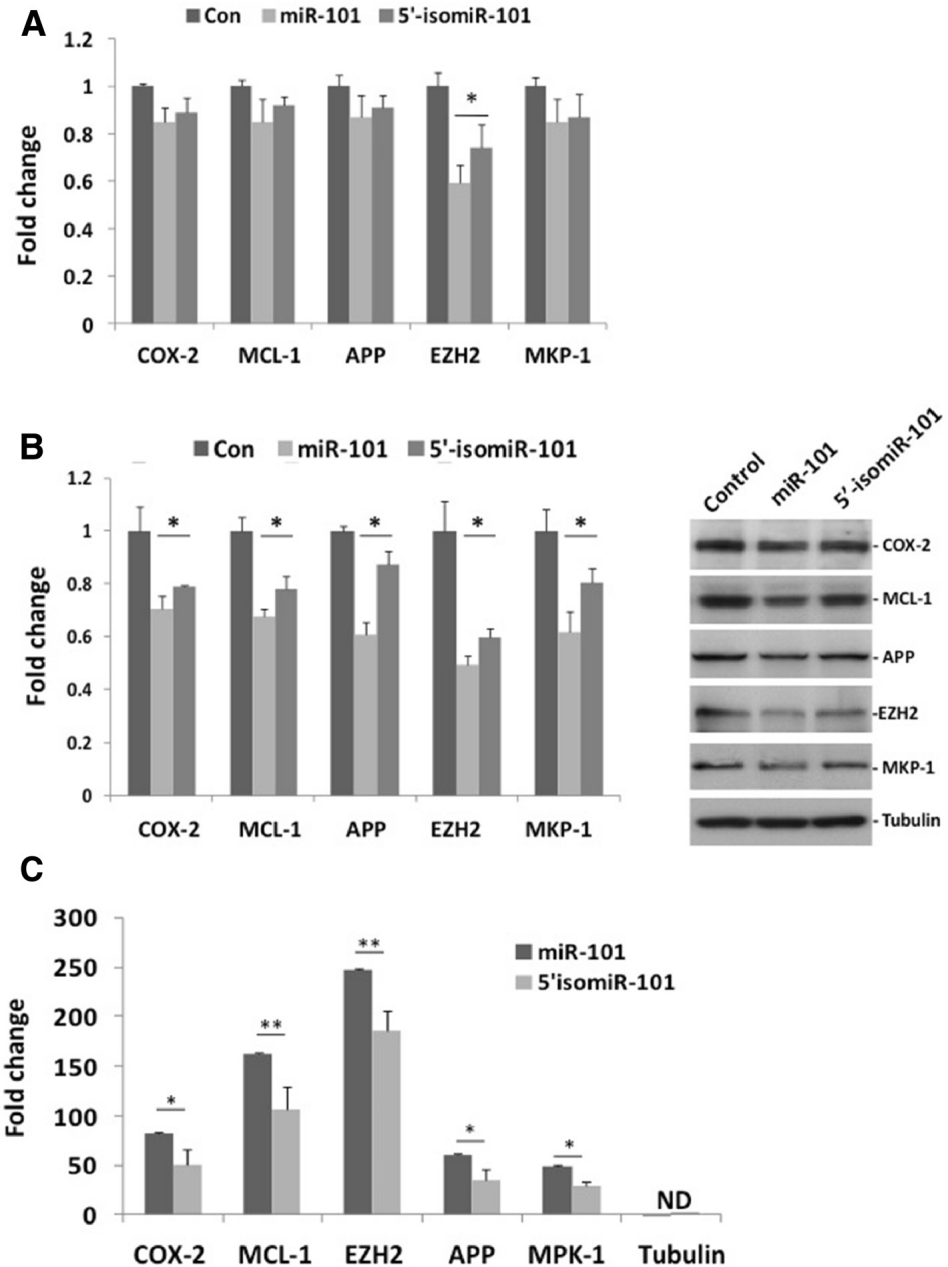
**miR-101 and 5’-isomiR-101 differentially regulate gene expression. A.** mRNA expression of miR-101 target genes following exogenous expression of miR-101 or 5’isomiR-101. After transfection of SH-SY5Y cells with the siGLO control sequence (Con) or the mIRIDIAN mimics for miR-101 or 5’-isomiR-101 the expression of COX-2, MCL-1, APP, EZH2 and DUSP-1 (MKP-1) was determined by RT-qPCRs. A control sample was used to normalize Fold Change values in each set of experiments. This control sample was assigned a value of 1. **B.** Protein expression of miR-101 target genes following exogenous expression of miR-101 or 5’isomiR-101. Western blot determination of COX-2, MCL-1, APP, EZH2 and DUSP-1 (MKP-1) levels in SH-SY5Y were performed in cells transfected with a control sequence (Con) and the mIRIDIAN mimics for miR-101 or 5’-isomiR-101. A control sample was used to normalize Fold Change values in each set of experiments. This control sample was assigned a value of 1. **C.** Expression of miR-101 target genes cells transfected with miR-101 and 5’-isomiR-101 biotynilated isomiRs. SH-SY5Y cells were transfected with Biotin, mIRIDIAN 3’-biotin-miR-101 or 3’-biotin-5’-isomiR-101. Biotin-miRNA complexes were pulled down with streptavidin beads and the expression of COX-2, MCL-1, APP, EZH2 and DUSP-1 (MKP-1) mRNA was determined by RT-qPCRs. Results are expressed as the Fold Change ratios obtained from the biotin-tagged miR-101 and 5’-isomiR-101 transfected cells versus the biotin transfected control cells. ND: Not detectable. All data are expressed as the mean ± SEM from at least three independent experiments. Asterisks indicate statistical significance between miR-101 and 5’isomiR-101 data: ** (p≤0.01), * (p≤0.5) using the Mann–Whitney test.

Several targets have been validated for miR-101, including the cyclooxygenase 2 (COX-2) [39], the myeloid leukemia cell differentiation protein (MCL-1) [38], the amyloid precursor protein (APP) [40], the enhancer of zeste homolog 2 (EZH2) [41] and the MAP kinase phosphatase 1 (DUSP1/MKP-1) [42]. Of these, DUSP1/MKP-1, APP and EZH2 mRNAs were predicted as possible targets of both miR-101 and 5’-isomiR-101. Transfection of SH-SY5Y cells with either miR-101 or 5’-isomiR-101 mimics slightly decreased mRNA levels of COX-2, MCL-1, APP and DUSP1 (MKP-1) compared with cells transfected with a scrambled-control sequence, although the differences observed were not statistically significant (Figure 3A). In contrast, miR-101 expression significantly decreased EZH2 mRNA levels, by 40%. A tendency for EZH2 down-regulation by miR-101 was also detected in the microarrays (fold change = −1.2; q = 15%). These findings corroborate previous results showing that miR-101 repressed the expression of certain genes at the protein level [38-40,42], while only EZH2 mRNA showed decay [41]. Our data indicate that, although similar amounts of miR-101 and 5’-isomiR-101 were delivered into neuronal cells, 5’-isomiR-101 inhibited EZH2 mRNA expression by 20%, suggesting that this variant is functional.

To further confirm 5’-isomiR-101 functionality we determined the effect of miR-101 and 5’-isomiR-101 mimics on the expression of COX-2, MCL-1, APP, EZH2 and MKP-1 at the protein level (Figure 3B). We found that comparable expression levels of exogenously transfected miR-101 or 5’-isomiR-101 in SH-SY5Y cells decreased the expression of all proteins. However, miR-101 produced a significantly higher down-regulation of the proteins studied compared with 5’-isomiR-101 (Figure 3B). In SH-SY5Y cells, miR-101 repressed the expression of COX-2, MCL-1, EZH2, APP and MKP-1 at the same levels as those reported in other cell systems [38-42].

The decreased silencing activity of 5’-isomiR-101 agrees with the reduced binding of this variant to Ago2 complexes. To further confirm that the target mRNAs were less represented in the RISC, we transfected SH-SY5Y cells with 3’biotinylated miR-101 and 5’-isomiR-101 mimics and subsequently pulled down the complexes with streptavidin. Both 3’biotinylated-miR-101 and 3’biotinylated-5’-isomiR-101 interacted with the mRNA of the five genes analyzed in the present study, although higher mRNA levels were found in the 3’biotinylated-miR-101 pulldowns (Figure 3C).

## Discussion

High-throughput sequencing has revealed the existence of miRNA sequence variants for virtually all miRNAs in many species and tissues and in both physiological and pathological conditions [16,43]. This scenario suggests a functional role for isomiRs that may introduce a new layer of complexity in gene expression regulation. Here, we characterized the functionality of a highly abundant type of miR-101 variant that differs in the seed region. miR-101 has mainly been studied for its tumor suppressive functions, by directly targeting EZH2, Cox-2, Mcl-1 and Fos [31,39,41,44-46]. miR-101 has also been shown to modulate the expression of target genes involved in inflammatory processes [42], autophagy [47] and angiogenesis [30]. In addition, our analysis showing the regulated expression of miR-101 in brain development and ageing and in a neurodegenerative process suggests important roles for this miRNA in nervous system physiology and pathology. Reinforcing this idea, genes that are relevant in brain function including APP [48] and ATX1 [49] are proven targets of miR-101.

For the majority of miRNAs, the most abundant isomiRs include 3’-trimming variants and nucleotide additions in the 3’-end [50], suggesting that modifications in the 3’ end are more permissive. In contrast, the diversity and abundance of isomiRs affecting the 5’-end of mature miRNAs (5’-trimming variants) is considerably reduced, which may be indicative of an increased impact of these variants in biological processes. In fact, miRNA 5’-trimming variants show a modified seed region (nucleotides 2–8 of the mature sequence) which is one of the major determinants defining target genes [50,51]. Thus, for a given miRNA, isomiRs expressed in high amounts which differ in the seed region are expected to have some impact on gene expression regulation. Our results show that among all the sequences mapping onto miR-101, 5’-trimming variants affecting a single nucleotide position are highly abundant in human samples and show variable expression depending on the sample. This suggests that the biogenesis and/or stability of miR-101 isomiRs are dependent on the cell context. 5’-trimming isomiRs may be generated by variations of the Dicer-cutting site on the precursor miRNA. In support of this idea, both types of miR-101 variants are strongly and similarly decreased (data not shown) when analyzing publicly available sRNA sequencing datasets in MCF-7 cells knocked down for Dicer [52]. Recent results indicate that Dicer activity can be modulated by a number of proteins, interacting either with Dicer or with miRNA precursors [53]. Thus, whether differential modulation of Dicer activity in diverse cell types and/or physiological conditions is underlying the relative abundance of 5’-trimming miR-101 isomiRs is an open question that deserves specific research.

Another possible mechanism accounting for the differential abundance of all variants mapping onto miR-101 may be related to the stability of the mature forms. It has been shown that the post-transcriptional addition of non-genome-encoded nucleotides to the 3’ end of mature miRNA increases miRNA stability or abundance [24,54]. However, the proportion of species with 3’-nucleotide additions mapping as miR-101 or 5’-isomiR-101 did not correlate with the differential miR-101/5’-isomiRs-101 relative abundance detected in the samples examined (data not shown). This suggests that nucleotide additions may not be a major explanation for the differential relative abundance.

Several findings indicate that miR-101 5’-trimming variants are functional silencing sRNA: i) both 5’-isomiR-101 mimic and endogenous 5’-isomiR-101 were loaded into Ago2 immunocomplexes, ii) biotin-streptavidin pull-down showed that 5’-isomiR-101 binds to both Ago2 and Rck/p54 RISC components iii) biotinylated 5’-isomiR-101 pull-down assays purified known miR-101 target mRNAs and iv) 5’-isomiR-101 mimic inhibited the expression of known miR-101 targets.

In trying to identify possible candidates that are differentially modulated by miR-101 and 5’-isomiR-101, high-throughput array-based approach failed to show significant regulation of mRNAs by either variant. It may be that miR-101 modulates gene expression mainly at the translational level, producing mild effects on RNA levels that are difficult to verify in a microarray approach. In spite of this, the arrays identified a small number of genes that were regulated by only one type of variant. In addition, the non-overlapping anti-correlation between the expression profiles of miR-101 variants and a number of age-related genes in the human brain agrees with specific miR-101- and 5’-isomiR-101 gene targeting. Although selective functions of the seed variants may add complexity to miR-101 regulatory effect, it has been proposed that isomiRs function cooperatively to target common biological pathways [55]. In agreement with this suggestion, the high number of commonly predicted targets indicates that similar biological pathways are regulated by isomiR-101 and 5’-isomiR-101.

A number of results suggest that 5’-isomiR-101 is less efficient than miR-101 in gene silencing: i) determinations of 5’-isomiR-101 and miR-101 with highly specific RT-PCR assays in IPs of endogenous Ago2 indicate that miR-101 is more efficiently loaded into the RISC, although the analysis of SH-SY5Y small RNA sequencing data suggests that 5’-isomiRs-101 are more abundant than the reference miR-101; ii) increased amounts of miR-101 were also detected in Ago2 IPs of SH-SY5Y cells transfected with equivalent quantities of miR-101 or 5’-isomiR-101 mimics; iii) biotinylated miR-101 bound increased amounts of both Ago2 and Rck/p54 RISC members compared with biotinylated 5’-isomiR-101 and iv) 5’-isomiR-101 showed a decreased ability to inhibit several miR-101 targets. Overall, these data indicate that the silencing activity of miR-101 variants is related to their efficient loading into the RISC and further suggest that the amount of the different miR-101 variants expressed within a cell does not strictly correlate with the quantities detected in the Ago2 complexes. Since Ago2 is the only Ago with mRNA slicing activity, decreased loading of 5’-isomiR-101 onto Ago2 may explain the less efficient gene silencing of at least some target genes showing RNA destabilization. However, the reduced loading of 5’-isomiR-101 in Ago1 and Ago3 detected in monocytic human cells suggests that less efficient binding to other Agos may contribute to the decreased silencing at the protein level. It has been shown that the 5’-terminal residue in a miRNA influences its loading into the RISC, with terminal 5’-U enhancing the Ago2 complex [56]. Thus, the 5’ terminal G in the isomiR may provide a less efficient incorporation into the RISC.

Our analysis suggests that the relative amount of the two main miR-101 seeds was not equivalent in the different Agos in monocytic human cells. It has recently been shown that a subset of miRNAs give rise to distinct isomiRs which associate mainly with distinct Ago proteins, both in plants [57] and in human samples [58,59], supporting an isomiR-dependent differential output in gene silencing.

## Conclusions

In summary, our data show that 5’-isomiR-101 is a functional silencing small RNA. We propose that in addition to specific gene targeting, 5’-isomiR-101 variants present a subtle effect through regulatory mechanisms involving decreased silencing efficiency. Changes in the relative expression of seeds ratios may provide scenarios for isomiR-selective qualitative and quantitative gene expression regulation. This may add complexity to the fine-tuned nature of miRNA regulation of gene expression.

The mechanisms by which miR-101 variants are generated and a full characterization of the repertoire of targets modulated by miR-101 and 5’-isomiR-101 may shed light on the respective biological function. Extensive study of these aspects will broaden our understanding of the overall biological impact of isomiRs in different tissues and physiological states.

## Methods

### Antibodies and reagents

Anti-Ago2 was from Millipore, anti-Rck/p54 was from MBL, anti-Mcl-1 was from Cell Signaling, anti-COX-2 was from Cayman, anti-EZH2 was obtained from Dr. Kristian Helllin (BRIC-University of Copenhagen), anti-MKP-1 was from Santa Cruz, anti-APP was from Abcam and anti-tubulin and anti-FLAG (M2) were from Sigma. miRIDIAN™ hsa-miRNA-101 mimics and siGLO Green (transfection indicator) were from Dharmacon: hsa-miRNA-101 (mature sequence: UACAGUACUGUGAUAACUGAA), hsa-5’-isomiR-101 (mature sequence: GUACAGUACUGUGAUAACUGA), 3’biotinilated hsa-miRNA-101 (mature sequence: UACAGUACUGUGAUAACUGAA-Bio) and 3’biotinilated hsa-5’-isomiR-101 (mature sequence: GUACAGUACUGUGAUAACUGA-Bio).

### Human samples

Brain samples of individuals without clinical neurological disease and no major histopathological lesions were obtained from the Institute of Neuropathology and the University of Barcelona Brain Bank, after the informed consent of the patients or their relatives and the approval of the local ethics committee had been given. Blood samples of healthy individuals without clinical neurological disease were obtained from the Hospital Clinic of Barcelona after written informed consent of the participants and approval of the local Ethics Committee had been given.

### RNA sequencing and miR-101 variability analysis

We used publicly available small RNA sequencing datasets from the GEO repository (GSE31069, GSM522374, GSE16579, GSE18069). In addition we sequenced small RNA in human blood samples (4 individuals), human brains (amygdala area of 4 individuals without major neurodegenerative histopathological signs) and SH-SY5Y neuroblastoma cells before and after differentiation to a postmitotic phenotype as previously described [60]. In these samples, total RNA was isolated using PAXgene blood RNA Kit (Qiagen) or the miRNAeasy kit (Qiagen). From each sample, 1μg of total RNA was used to prepare libraries according to the “TruSeq Small RNA Sample Preparation Guide” (Part# 15004197 Rev. A). Libraries were subsequently sequenced on one Illumina Genome Analyzer II run using 36 single-read cycles. For processing of the sequencing data (recognition and removal of the adapter) and characterization of miRNA variants we used a stand-alone version of the Seqbuster tool (http://estivill_lab.crg.es/seqbuster[50]). The parameters were set up to detect isomiRs by: a) trimming at the 3’/5’-end (up to 3 nucleotides shifted from the reference dicing site), b) addition at the 3-end (up to 3 nucleotides added) and c) nucleotide substitution (up to 1 mismatch with respect to the reference miRNA sequence).

### miR-101/ age-related genes expression-correlation studies in the human brain

To identify the genes differently expressed in the brain at different ages we applied the same method described by Somel et al. [26]. Briefly, the intensity of gene expression at 12 different time-points along life was used to correlate to age. Expression was fitted to 8 non-linear functions capable of detect linear, quadratic or more complex correlation between gene expression levels and age. All genes that fitted to one of those 8 models with p-value lower than 0.01 were considered age-related genes.

We considered an age-related gene to be targeted by miR-101 or 5’-isomiR-101 sequences only if: i) there is a pair complementary between the miRNA seed and the gene according to TargetScan Custom prediction algorithm [37] and ii) there is a negative expression correlation between the miRNA sequence and the gene. Only those genes that have been detected in the gene expression profile of the brain samples, according to the public data from the original work, were used as input for TargetScan Custom algorithm. For each miR-101-sequence-gene pair predicted by TargetScan Custom, a linear regression model was performed by the standard R function (’lm’). To consider a gene regulated by a miR-101 variant, the p-value of the model should be less than 0,01 (F-test) and Pearson’s product moment correlation lower than −0.70. Normalized intensities of microarray data for gene expression, and logarithms of counts for miRNA sequences were used. The numbers of assigned miR-101 or 5’-isomiR-101 predicted targets were calculated according to the first requirement (seed match) or both of them (seed match and anti-correlation). The statistical differences between the numbers of commonly predicted miR-101 and 5’-isomiR-101 targets, considering or not the anti-correlation criterion was determined with the prop.test function in R.

### Cell cultures

SH-SY5Y and SH-SY5Y cells stably expressing Ago2-FLAG (SH-SY5Y-Ago2-FLAG) [61] were cultured at 37°C in a 95:5 air/CO2 water saturated atmosphere in 1:1 mixture of DMEM and Ham’s F12 medium and 10% supplemental fetal bovine serum. For cell transfection, SH-SY5Y cells were seeded at 0.2 × 10^6^ and 50 ng of hsa-miRNA mimic or SiGLO Green (Control) were transfected with Lipofectamine Plus according to the manufacturer’s instructions. For western-blotting, 48 h post-transfection the medium was removed and the cells were washed twice with cold phosphate-buffered saline (PBS) and lysed with Lysis Buffer (50 mM Tris–HCl, pH 7.5, 150 mM NaCl, 1.5 mM MgCl_2_, 1.5 mM EGTA, 1 mM EDTA, 1% Triton X-100, 1 mM DTT, and Protease and Phosphatase Inhibitors). After 15 min on ice, the lysate was removed from the dishes and centrifuged at 12,000 *g* for 20 min at 4°C. Supernatants were quantified using Bradford method and kept until use. For RNA analysis cells were harvested and treated as described above.

### Affinity purification of miRNA complexes

Biotin-tagged miRNA complexes were purified as described before [62]. Briefly, transfected SH-SY5Y cells were harvested 48 h after transfection, washed in PBS and scraped in 1 ml of lysis buffer (20 mM Tris; pH 7.5, 200 mM NaCl, 2.5 mM MgCl_2_, 0.05% Igepal, 60 U Superase-In/ml (Ambion), 1 mM DTT and Pefabloc (Roche). Lysate was vortexed twice for 20 seconds and cleared by spinning at 12,000*g* at 4 °C for 20 min in a microcentrifuge. Fifty μl of the sample were saved for input analysis and placed on dry ice. The remaining lysate was incubated for 1 h at 4 °C rotating with 25 μl pre-washed streptavidin-sepharose beads. In order to prevent non-specific binding of RNA and protein complexes, the beads were coated with RNase-free BSA and yeast tRNA (both from Ambion). Beads were washed three times with 500 μl ice-cold lysis buffer. In order to isolate the RNA, TRIzol was added to both input and affinity-washed beads. 40 μl of chloroform was added and after shaking samples were spin at 12,000 *g* at 4 °C for 15 min in a microcentrifuge. Supernatant was transferred to a fresh tube and RNA was precipitated adding 1 μl of GlycoBlue co-precipitant (Ambion), 6 μl of 5 M NaCl and 360 μl of ice-cold 95% ethanol overnight at −20 °C. Samples were pelleted at 12,000*g* for 15 min at 4°C, washed in 70% ethanol in RNase-free water and air-dried. RNA was dissolved in 120 μl of RNase-free water (Ambion). In order to isolate the proteins bound to the biotynilated miRNAs, washed beads were resuspended in SDS-buffer and boiled at 100°C.

The microRNA fraction from human frontal cortex lysate was purified using the microRNA isolation kit from Wako based on Ago2 immunoprepipitation complexes, following manufacturer’s instructions.

### Western-blot

The protein content of either immunoprecipitations or cellular extracts were run on 8%-12% SDS-PAGE gels, transferred on to PVDF membranes, and incubated with the corresponding antibodies at the following concentrations: Ago-2 1:2000; Rck/p54: 1:1000; Mcl-1: 1:1000; COX-2: 1:500; EZH2: 1:1000; MKP-1: 1:250; APP: 1:1000; Tubulin: 1:10.000. The membranes were developed with the enhanced chemiluminescence method (ECL) following the manufacturer’s instructions.

### mRNA expression arrays

SH-SY5Y cells were transfected with miR-101 or 5’-isomiR-101 as previously described in three independent experiments, and total RNA was isolated 48 h later. RNA concentration was measured with a Nanodrop™, and RNA quality was assessed by Bioanalyzer™ with RIN (RNA integrity number) ranging between 9 and 10. For each sample, 200 ng of total RNA was reverse transcribed, amplified by *in vitro* transcription and labeled with biotin-UTP using the Illumina Total Prep RNA amplification kit (IL1791, Applied Biosystem/Ambion, Austin, TX, USA) following the manufacturer’s instructions. 750 ng of biotinylated cRNA was hybridized in a BeadChip Hyb Chamber with rocking for 16 h at 58°C. Bead arrays were washed and blocked in a rocking incubator. Finally, bead arrays were dried by centrifugation followed by scanning in the Illumina Beadstation. The raw data were summarized per probe using BeadStudio software Gene Expression. The SAM (significance analysis of microarrays) two-class unpaired comparison test was applied to detect significant differences in gene expression between treated and control conditions, initially setting the statistical significance at a false discovery rate of 5%, with an arbitrary absolute fold chance cutoff set at 1.2.

### RT-qPCR

#### miRNA-RT-qPCR

Quantitative real time PCR was performed using the miRCURY LNA™ microRNA PCR System (Exiqon) on total RNA extracted from SH-SY5Y with mirVana’s isolation kit (Ambion) following the manufacturer’s instructions. PCR amplification and detection were performed with the ROCHE LightCycler 480 detector, using 2x SYBR GREEN Master Mix. The reaction profile was: Polymerase Activation/Denaturation cycle (95°C for 10 min) followed by 40 amplification cycles (95°C-10”, 60°C-20”). Each sample was amplified in triplicate. miRNA levels were calculated using the LightCycler 480 software. Both problem and calibrator samples were normalized by the relative expression of reference small non-coding RNAs (SNORD44 and SNORD48).

#### mRNA RT-qPCR

Total RNA was extracted from SH-SY5Y cells with mirVana isolation kit (Ambion). Purified RNAs were treated with RNase-free DNAse (DNA-free, Ambion) and reverse-transcribed, with Superscript II (Invitrogen) to generate the corresponding cDNAs that served as PCR templates for mRNA quantification. The primers used for RT-qPCR were (5’->3’): COX-2; F-CCTGTGCCTGATGATTGC, R-CTGATGCGTGAAGTGCTG, MCL-1; F-AAAGAGGCTGGGATGGGTTT, R-CAAAAGCCAGCAGCACATTC, APP; F-TCAGGTTGACGCCGCTGT, R-TTCGTAGCCGTTCTGCTGC, EZH2; F-AGTGTGACCCTGACCTCTGT, R-AGATGGTGCCAGCAATAGAT, DUSP-1 F-CAGCTGCTGCAGTTTGAGTC, R- AGAGGTCGTAATGGGGCTCT. PCR amplification and detection were performed with the ROCHE LightCycler 480 detector, using 2X SYBR GREEN Master Mix as reagent following the manufacturer’s instructions. The reaction profile was: denaturation/activation cycle (95° for 10 min) followed by 40 cycles of denaturation-annealing-extension (95°-10’, 60°-40”, 72°-10”) and a final melting cycle (95°-5’, 72°-10”, 98°-continuous). Each sample was amplified in triplicate. mRNA levels were calculated using the LightCycler 480 software. Samples were normalized by the relative expression of housekeeping gene (Tubulin).

#### Statistical methods

Statistical analysis was performed with G-STAT software (http://www.gstat.org). The Mann–Whitney non-parametric test was used for the statistical analysis (two-tailed) of the RT-qPCR and western blot assays in transfection experiments; *P≤* 0.05 was considered statistically significant.

In Figures 2A and 3C, results are expressed as the mean of the Fold Change ratios (microRNA - Biotin-mRNA / control siGLO - Biotin) ± SEM. In Figure 2B and 2D, results are expressed as the mean of the Fold Change ratios (FLAG-Ago2 Antibodies / control Antibodies) ± SEM. In Figure 2C, a miRNA-101 transfected sample was used to normalize data and assigned a value of 1 (Arbitrary Units). In Figure 3A and 3B, a control sample was used to normalize Fold Change values in each set of experiments and SEM was calculated. This control sample was assigned a value of 1.

## Abbreviations

miR: MicroRNA; HD: Huntington Disease; RISC: RNA induced silencing complex.

## Competing interests

The authors have declared that no competing interests exist.

## Authors’ contributions

FLL carried out the molecular studies, participated in the experimental design and data analysis and drafted the manuscript. MBC performed molecular studies and participated in the experimental design. JAR and IFA participated in experimental design and manuscript discussion. LP performed the bioinformatics analyses. XE participated in the coordination of the study. EM conceived of the study, participated in the design, data analysis and coordination and helped to draft the manuscript. All authors read and approved the final manuscript.

## Supplementary Material

Additional file 1: Figure S1 miR-101 variants in the human frontal cortex. The different sequences annotating to miR-101, the respective frequencies and lengths are shown. For the 5’ and 3’ trimming variants, the number of nucleotides upstream (up) or downstream (down) of the reference miRBase miR-101 (highlighted in blue), are shown. The nucleotides involved in the 5’- and 3’ variant are next indicated. In 3’-addition variants, the number and type of nucleotides added to the 3’-ends are shown. In the nucleotide substitution variants, the affected position is first indicated; the pair of nucleotides that follow next indicates the substituted and the original nucleotides, respectively. Only variants with more than 10 counts are shown. The more abundant sequences mapping onto miR-101 locus that represent the reference miR-101 sequence (in blue) and the 5’-isomiR-101 (in red) contained in a grey box are the chosen sequences for functional assays in transfection Click here for file

Additional file 2: Table S1 miR-101 variants in the human striatum, amigdala and frontal gyrus (Somel et al., 2010) brain areas. The different sequences annotating to miR-101, the respective frequencies and lenghts are shown. For the 5’ and 3’ trimming variants, the number of nucleotides upstream (up) or downstream (down) of the reference miRBase miR-101, are shown. The nucleotides involved in the 5’- and 3’ variant are next indicated. In 3’-addition variants, the number and type of nucleotides added to the 3’-end are shown. In the nucleotide substitution variants, the affected position is first indicated; the pair of nucleotides that follow next indicates the substituted and the original nucleotides, respectively. Only variants with more than 10 counts were considered. The sequences mapping onto miR-101 locus that represent the reference miR-101 sequence (in blue) and the 5’-isomiR-101 (in red) contained in a grey box. Click here for file

Additional file 3: Figure S2 miR-101 and 5’isomiR-101 frequency distribution in different Agos. A. Normalized expression levels of all sequences mapping onto miR-101 (blue bars), 5’-isomiR-101 seed (red bars) and reference miR-101 seed (green bars) in Ago1-Ago3 IP and in total cell extracts (Total). B. Table showing several determinations of miR-101 sequences. Freq. indicates the total count number; Number of variants indicates the sequence diversity for mIR-101; Norm. Freq., indicates the normalized frequency calculated as freq. miR-101/freq. mIRNAs *10E6. Click here for file

Additional file 4: Table S2 Genes differently expressed after transfection with isomiR-101 mimic or a scrambeled (scr) sequence. The threshold for significant regulation is considered as a variation in the expression fold change above 1,2 or below -1,2 and a false discovery rate below 5% (q<5), whch is indicated in bold. The MiRWalk database on predicted and validated microRNA targets (http://www.umm.uni-heidelberg.de/apps/zmf/mirwalk/index.html) was used to highlight additional information: i) The prediction of the deregulated genes as putative targets for miR-101 by different algorithms (-, indicates no prediction and na information not available) and ii) the presense of putative miR-101 target seed sites in regions other than the 3’-UTR (-, indicates no sites and na information not available). In the last column, the custom Targetscan 5.2 prediction algorithm was used to highlight putative 5’-isomiR-101 targets. Click here for file

Additional file 5: Table S3 Genes differently expressed after transfection with 5’-isomiR 101 mimic or a scrambeled (scr) sequence. The threshold for significant regulation is considered as a variation in the expression fold change above 1,2 or below -1,2 and a false discovery rate below 5% (q<5), whch is indicated in bold. The MiRWalk database on predicted and validated microRNA targets (http://www.umm.uni-heidelberg.de/apps/zmf/mirwalk/index.html) was used to highlight additional information: i) The prediction of the deregulated genes as putative targets for miR-101 by different algorithms (-, indicates no prediction and na information not available) and ii) the presense of putative miR-101 target seed sites in regions other than the 3’-UTR (-, indicates no sites and na information not available). In the last column, the custom Targetscan 5.2 prediction algorithm was used to highlight putative 5’-isomiR-101 targets. Click here for file

Additional file 6: Figure S3 Anti-correlation of age-related genes and miR-101 and 5’-isomiR-101 expression profiles. A. Distribution of the numbers and percentages of age-related genes (blue) targeted by miR-101 (red) and 5’-IsomiR-101 (green) seeds, according to TargetScan algorithm. B. Expression profile of the more abundant miR-101 and 5’-isomiR-101 sequences, and two example age-related genes. NYAP2 expression anti-correlated with that of 5’-isomiR-101, and SCN3B expression anti-correlated with that of miR-101 (considering an anti-correlation threshold < −0,7). Click here for file
