# Supplementary material for: A highly expressed miR-101 isomiR is a functional silencing small RNA
Source: BMC Genomics. 2013 Feb 15;14:104. doi: 10.1186/1471-2164-14-104 (PMC3751341; doi:10.1186/1471-2164-14-104)
Supplement: Additional file 1: Figure S1 — miR-101 variants in the human frontal cortex. The different sequences annotating to miR-101, the respective frequencies and lengths are shown. For the 5’ and 3’ trimming variants, the number of nucleotides upstream (up) or downstream (down) of the reference miRBase miR-101 (highlighted in blue), are shown. The nucleotides involved in the 5’- and 3’ variant are next indicated. In 3’-addition variants, the number and type of nucleotides added to the 3’-ends are shown. In the nucleotide substitution variants, the affected position is first indicated; the pair of nucleotides that follow next indicates the substituted and the original nucleotides, respectively. Only variants with more than 10 counts are shown. The more abundant sequences mapping onto miR-101 locus that represent the reference miR-101 sequence (in blue) and the 5’-isomiR-101 (in red) contained in a grey box are the chosen sequences for functional assays in transfection [file 1471-2164-14-104-S1.pdf]

Figure 1

Sequence	Frequency	Lenght	5'-Trimm. variants	3'-Trimm. variants	3'-Add. variants	3'-Nt Subtit. variants
ACAGUACUGUGAUAACUGA	38	19	1-down A	1-up A	-	-
ACAGUACUGUGAUAACUGAA	43	20	1-down A	-	-	-
ACAGUACUGUGAUAACUGAAA	20	21	1-down A	-	1-A	-
ACAGUACUGUGAUAACUGAAG	13	21	1-down A	1-down G	-	-
AUACAGUACUGUGAUAACUGA	14	21	1-up G	1-up A	-	1AG
GCACAGUACUGUGAUAACUGA	11	21	1-up G	1-up A	-	2CU
GGACAGUACUGUGAUAACUGA	41	21	1-up G	1-up A	-	2GU
GGACAGUACUGUGAUAACUGAA	26	22	1-up G	-	-	2GU
GGACAGUACUGUGAUAACUGAAA	11	23	1-up G	-	1-A	2GU
GUAAAGUACUGUGAUAACUGA	14	21	1-up G	1-up A	-	4AC
GUAAAGUACUGUGAUAACUGAA	19	22	1-up G	-	-	4AC
GUACAGGACUGUGAUAACUGA	15	21	1-up G	1-up A	-	7GU
GUACAGGACUGUGAUAACUGAA	14	22	1-up G	-	-	7GU
GUACAGUAAUGUGAUAACUGA	36	21	1-up G	1-up A	-	9AC
GUACAGUAAUGUGAUAACUGAA	24	22	1-up G	-	-	9AC
GUACAGUACUGGGAUAACUGA	20	21	1-up G	1-up A	-	12GU
GUACAGUACUGGGAUAACUGAA	17	22	1-up G	-	-	12GU
GUACAGUACUGUGAGAACUGAA	10	22	1-up G	-	-	15GU
GUACAGUACUGUGAUAACU	906	19	1-up G	3-up GAA	-	-
GUACAGUACUGUGAUAACUA	43	20	1-up G	3-up GAA	1-A	-
GUACAGUACUGUGAUAACUAA	35	21	1-up G	3-up GAA	2-AA	-
GUACAGUACUGUGAUAACUAAA	12	22	1-up G	3-up GAA	3-AAA	-
GUACAGUACUGUGAUAACUG	519	20	1-up G	2-up AA	-	-
GUACAGUACUGUGAUAACUGA	7474	21	1-up G	1-up A	-	-
GUACAGUACUGUGAUAACUGAA	5766	22	1-up G	-	-	-
GUACAGUACUGUGAUAACUGAAA	1813	23	1-up G	-	1-A	-
GUACAGUACUGUGAUAACUGAAAA	380	24	1-up G	-	2-AA	-
GUACAGUACUGUGAUAACUGAAAAA	79	25	1-up G	-	3-AAA	-
GUACAGUACUGUGAUAACUGAAG	16	23	1-up G	1-down G	-	-
GUACAGUACUGUGAUAACUGAAU	20	23	1-up G	-	1-U	-
GUACAGUACUGUGAUAACUGAC	72	22	1-up G	1-up A	1-C	-
GUACAGUACUGUGAUAACUGAU	49	22	1-up G	1-up A	1-U	-
GUACAGUACUGUGAUAACUGU	202	21	1-up G	2-up AA	1-U	-
GUACAGUACUGUGAUAACUGUA	25	22	1-up G	2-up AA	2-UA	-
GUACAGUACUGUGAUAACUGUAA	13	23	1-up G	2-up AA	3-UAA	-
GUACAGUACUGUGAUAACUGUU	14	22	1-up G	2-up AA	2-UU	-
GUACAGUACUGUGAUAACUUA	24	21	1-up G	3-up GAA	2-UA	-
GUACAGUACUGUGAUAAUUGA	13	21	1-up G	1-up A	-	18UC
GUACAGUACUGUGAUAAUUGAA	11	22	1-up G	-	-	18UC
GUACAGUACUGUGAUACCUGA	48	21	1-up G	1-up A	-	17CA
GUACAGUACUGUGAUACCUGAA	27	22	1-up G	-	-	17CA
GUACAGUACUGUGAUACCUGAAA	11	23	1-up G	-	1-A	17CA
GUACAGUACUGUGAUCACUGA	18	21	1-up G	1-up A	-	16CA
GUACAGUACUGUGAUCACUGAA	10	22	1-up G	-	-	16CA
GUACAGUACUUUGAUAACUGA	16	21	1-up G	1-up A	-	11UG
GUACAGUACUUUGAUAACUGAA	17	22	1-up G	-	-	11UG

GUACAGUAUUGUGAUAACUGA	24	21	1-up G	1-up A	-	9UC
GUACAGUAUUGUGAUAACUGAA	24	22	1-up G	-	-	9UC
GUAGAGUACUGUGAUAACUGA	11	21	1-up G	1-up A	-	4GC
GUAGAGUACUGUGAUAACUGAA	11	22	1-up G	-	-	4GC
UAAAGUACUGUGAUAACUGA	17	20	-	1-up A	-	3AC
UAAAGUACUGUGAUAACUGAA	20	21	-	-	-	3AC
UAAAGUACUGUGAUAACUGAAA	10	22	-	-	1-A	3AC
UACAGGACUGUGAUAACUGA	13	20	-	1-up A	-	6GU
UACAGGACUGUGAUAACUGAA	22	21	-	-	-	6GU
UACAGGACUGUGAUAACUGAAA	13	22	-	-	1-A	6GU
UACAGUAAUGUGAUAACUGA	35	20	-	1-up A	-	8AC
UACAGUAAUGUGAUAACUGAA	44	21	-	-	-	8AC
UACAGUAAUGUGAUAACUGAAA	20	22	-	-	1-A	8AC
UACAGUACUGGGAUAACUGA	57	20	-	1-up A	-	11GU
UACAGUACUGGGAUAACUGAA	51	21	-	-	-	11GU
UACAGUACUGGGAUAACUGAAA	28	22	-	-	1-A	11GU
UACAGUACUGUGAUAAAUGA	16	20	-	1-up A	-	17AC
UACAGUACUGUGAUAAAUGAA	16	21	-	-	-	17AC
UACAGUACUGUGAUAACUA	16	19	-	3-up GAA	1-A	-
UACAGUACUGUGAUAACUG	356	19	-	2-up AA	-	-
UACAGUACUGUGAUAACUGA	6656	20	-	1-up A	-	-
UACAGUACUGUGAUAACUGAA	6867	21	-	-	-	-
UACAGUACUGUGAUAACUGAAA	3261	22	-	-	1-A	-
UACAGUACUGUGAUAACUGAAAA	606	23	-	-	2-AA	-
UACAGUACUGUGAUAACUGAAAAA	195	24	-	-	3-AAA	-
UACAGUACUGUGAUAACUGAAC	25	22	-	-	1-C	-
UACAGUACUGUGAUAACUGAACAA	18	24	-	-	3-CAA	-
UACAGUACUGUGAUAACUGAAG	1492	22	-	1-down G	-	-
UACAGUACUGUGAUAACUGAAGA	168	23	-	2-down GA	-	-
UACAGUACUGUGAUAACUGAAGAA	85	24	-	3-down GA	-	-
UACAGUACUGUGAUAACUGAAGU	31	23	-	1-down G	1-U	-
UACAGUACUGUGAUAACUGAAU	369	22	-	-	1-U	-
UACAGUACUGUGAUAACUGAAUAA	32	24	-	-	3-UAA	-
UACAGUACUGUGAUAACUGAC	61	21	-	1-up A	1-C	-
UACAGUACUGUGAUAACUGACC	28	22	-	1-up A	2-CC	-
UACAGUACUGUGAUAACUGACU	22	22	-	1-up A	2-CU	-
UACAGUACUGUGAUAACUGAU	23	21	-	1-up A	1-C	-
UACAGUACUGUGAUAACUGAUU	11	22	-	1-up A	2-UU	-
UACAGUACUGUGAUAACUGU	94	20	-	2-up AA	1-C	-
UACAGUACUGUGAUAAUUGA	15	20	-	1-up A	-	7UC
UACAGUACUGUGAUACCUGAA	15	21	-	-	-	6CA
UACAGUACUGUGAUCACUGA	13	20	-	1-up A	-	5CA
UACAGUACUGUGAUCACUGAA	17	21	-	-	-	5CA
UACAGUACUGUUAUAACUGA	10	20	-	1-up A	-	2UG
UACAGUACUGUUAUAACUGAA	12	21	-	-	-	2UG
UACAGUACUUUGAUAACUGA	11	20	-	1-up A	-	1-UG
UACAGUAGUGUGAUAACUGA	14	20	-	1-up A	-	8GC
UACAGUAGUGUGAUAACUGAA	15	21	-	-	-	8GC
UACAGUAGUGUGAUAACUGAAA	14	22	-	-	1-A	8GC
UACAGUAUUGUGAUAACUGAA	15	21	-	-	-	8UC

UACAUUACUGUGAUAACUGA	14	20	-	1-up A	-	5UG
UACAUUACUGUGAUAACUGAA	13	21	-	-	-	5UG
UCCAGUACUGUGAUAACUGA	11	20	-	1-up A	-	2CA
UCCAGUACUGUGAUAACUGAA	11	21	-	-	-	2CA
