# Supplementary figures and images for: A highly expressed miR-101 isomiR is a functional silencing small RNA
Source: BMC Genomics. 2013 Feb 15;14:104. doi: 10.1186/1471-2164-14-104 (PMC3751341; doi:10.1186/1471-2164-14-104)

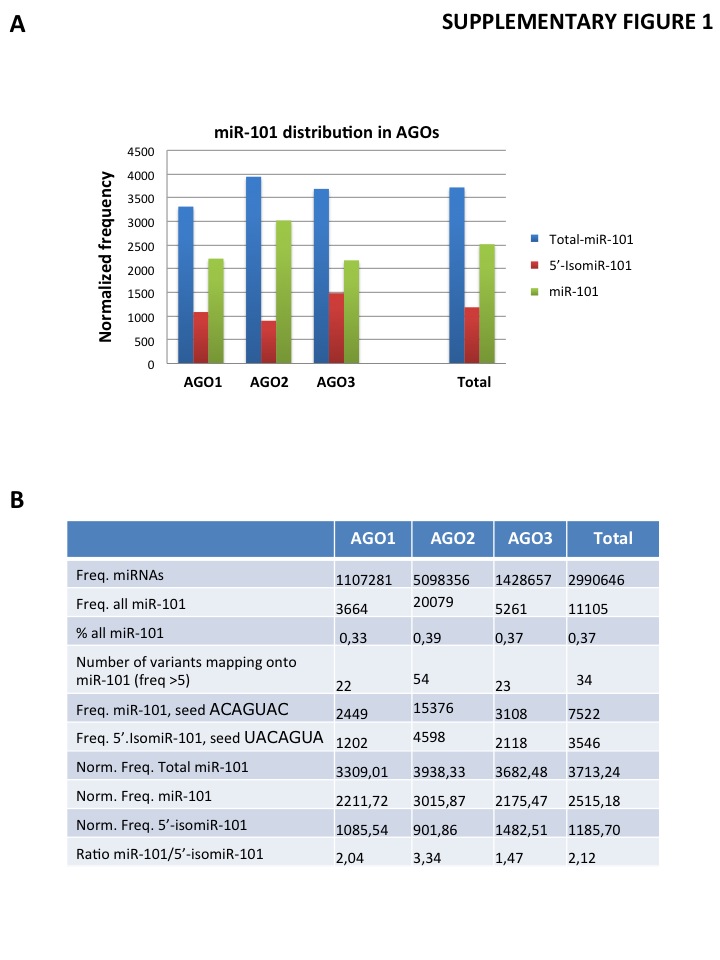

Supplement: Additional file 3: Figure S2 — miR-101 and 5’isomiR-101 frequency distribution in different Agos. A. Normalized expression levels of all sequences mapping onto miR-101 (blue bars), 5’-isomiR-101 seed (red bars) and reference miR-101 seed (green bars) in Ago1-Ago3 IP and in total cell extracts (Total). B. Table showing several determinations of miR-101 sequences. Freq. indicates the total count number; Number of variants indicates the sequence diversity for mIR-101; Norm. Freq., indicates the normalized frequency calculated as freq. miR-101/freq. mIRNAs *10E6. [file 1471-2164-14-104-S3.jpeg]

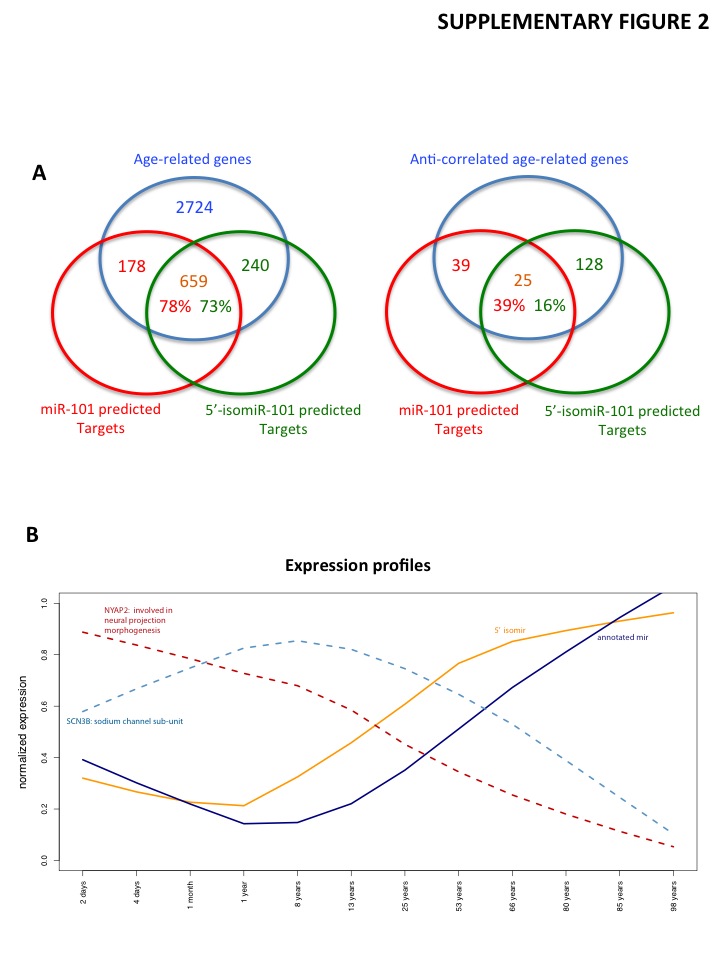

Supplement: Additional file 6: Figure S3 — Anti-correlation of age-related genes and miR-101 and 5’-isomiR-101 expression profiles. A. Distribution of the numbers and percentages of age-related genes (blue) targeted by miR-101 (red) and 5’-IsomiR-101 (green) seeds, according to TargetScan algorithm. B. Expression profile of the more abundant miR-101 and 5’-isomiR-101 sequences, and two example age-related genes. NYAP2 expression anti-correlated with that of 5’-isomiR-101, and SCN3B expression anti-correlated with that of miR-101 (considering an anti-correlation threshold < −0,7). [file 1471-2164-14-104-S6.jpeg]
